# Biotechnological Screening of Microalgal and Cyanobacterial Strains for Biogas Production and Antibacterial and Antifungal Effects 

**DOI:** 10.3390/metabo4020373

**Published:** 2014-05-15

**Authors:** Opayi Mudimu, Nataliya Rybalka, Thorsten Bauersachs, Jens Born, Thomas Friedl, Rüdiger Schulz

**Affiliations:** 1 Botanical Institute, Department of Plant Cell Physiology and Biotechnology, Christian-Albrechts- University of Kiel, Am Botanischen Garten 5, D-24118 Kiel, Germany; E-Mails: Nataliya.Rybalka@biologie.uni-goettingen.de (N.R.); rschulz@bot.uni-kiel.de (R.S.); 2 Albrecht-von-Haller-Institute, Department of Experimental Phycology and Culture Collection of Algae (SAG), Georg August University of Göttingen, Untere Karspüle 2, 37073 Göttingen, Germany; E-Mail: tfriedl@uni-goettingen.de; 3 Institute of Geosciences, Department of Organic Geochemistry, Christian-Albrechts-University of Kiel, Ludewig-Meyn-Str. 10, D-24118 Kiel, Germany; E-Mail: thb@gpi.uni-kiel.de; 4 Institute for Chemical Technology, Flensburg University of Applied Science, Kanzleistr. 91-93, D-24943 Flensburg, Germany; E-Mail: jens.born@fh-flensburg.de

**Keywords:** microalgae, cyanobacteria, biomass and biogas production, antibacterial and antifungal effects

## Abstract

Microalgae and cyanobacteria represent a valuable natural resource for the generation of a large variety of chemical substances that are of interest for medical research, can be used as additives in cosmetics and food production, or as an energy source in biogas plants. The variety of potential agents and the use of microalgae and cyanobacteria biomass for the production of these substances are little investigated and not exploited for the market. Due to the enormous biodiversity of microalgae and cyanobacteria, they hold great promise for novel products. In this study, we investigated a large number of microalgal and cyanobacterial strains from the Culture Collection of Algae at Göttingen University (SAG) with regard to their biomass and biogas production, as well antibacterial and antifungal effects. Our results demonstrated that microalgae and cyanobacteria are able to generate a large number of economically-interesting substances in different quantities dependent on strain type. The distribution and quantity of some of these components were found to reflect phylogenetic relationships at the level of classes. In addition, between closely related species and even among multiple isolates of the same species, the productivity may be rather variable.

## 1. Introduction

### 1.1. Microalgae Biomass as Substrate in Biogas Plants

Energy is increasingly becoming a scarce resource worldwide. Fossil-based fuels (oil, gas and coal) are presently the most affordable energy source but will increasingly become expensive as these resources will rapidly decrease with growing global demand in the future. The use of fossil-based fuel has also led to an increase in the concentration of greenhouse gases in the atmosphere with severe perturbations of the Earth’s global climate [1]. Renewable energy has been pointed out as an interesting alternative to fossil fuels due to significantly reduced air pollution. Motivated by the need to meet the ever increasing energy demand and sustainability consciousness, renewable energy has been promoted technologies, such as wind energy, solar energy, and biomass production. Bioenergy can be produced from a very wide range of agricultural biomass as well as from a range of crop plants [2]. Suitable agricultural substrates for the production of biomass fuels are, for example, maize, sugar beets, and clover grass [3]. In general, the use of biomass fuel will not contribute to global warming as the carbon dioxide released by burning equals the amount absorbed from the atmosphere during the plant growth [4].

Biomass can be converted into biogas by anaerobic fermentation. The latter is a process that enzymatically converts organic substance into biogas and digestate. The use of crop plants for biogas production is undesired because of the competition with food and feed production and the use of arable land. For these reasons animal manures, crop residues, and organic wastes have attracted much attention as feedstock for the production of biogas [5,6]. One possible alternative for biogas production is production of biomass by microalgae. During this process, sun energy is converted into biological energy that is contained in algal biomass. Compared to crop plants the production of biomass by microalgae offers numerous advantages because of the capability to grow throughout the year, noncompetitive cultivation on arable land and the use of sea water for marine species precludes the necessity to employ fresh water. Microalgae are suitable for cultivation at high CO_2_ concentrations [7,8,9], as well as to convert CO_2_ from flue gas into biomass and subsequently biomass to biofuels and other valuable products [10]. In addition, their growth rates are higher than conventional crop plants under optimal culture condition [11,12]. One drawback in the utilization of microalgae biomass as substrate in biogas plants is the lower production capacity and actual higher costs for their cultivation compared to the production of biomass from maize and sugar beets. Anaerobic fermentation of algae biomass was first investigated in the 1950s [13]. The variety of potential agents and the use of different microalgae species for anaerobic fermentation are little investigated and not exploited for the current economic markets.

Multiple research projects have been performed to investigate the influence of various environmental parameters on the anaerobic fermentation of algal strains, as well as the pretreatment of microalgae biomass in order to increase the anaerobic biodegradability. The suitability of six microalgae species was investigated in order to integrate anaerobic fermentation in final step of microalgae-based biorefinery concepts [14]. These authors showed that the biogas potential is strongly dependent on the species and on the pretreatment. Likewise, Ras *et al.* [15] studied methane production from a coupled production—digestion unit of microalgae under two hydraulic retention times. A life-cycle assessment of biogas production from the microalgae *Chlorella vulgaris* was performed. The results were compared to algal biodiesel and to first generation biodiesels and showed that the impacts generated by the production of methane from microalgae are strongly correlated with the electric consumption [16]. These authors demonstrated that production, harvesting, and concentration of algae and their transformation to methane represent a high-energy debt (electric consumption) for methane production process from algae. Sialve *et al.* [17] proposed the theoretical methane yield based on microalgae biochemical composition and reported the strategies to improve their conversion into methane. The screening of a large number of microalgae and cyanobacteria, however, has yet not been undertaken.

Microalgae as well as cyanobacteria differ significantly due to their morphological, biochemical, and physiological composition. Classification is based on and reflects differences of composition (especially pigments). For the anaerobic fermentation, the same composition, such as structure of cell wall, plays a decisive role for the degradability of biomass, as well as the production of biogas.

### 1.2. Distribution of Compounds with Bioactive Activities

The evolution of bacterial resistance to antibiotics has been known over recent years and is induced by several mechanisms such as the transfer of antibiotic resistance genes between bacteria [18]. The Effectiveness of many known antibiotics gradually decreases. At the same time the antibiotic resistance increases continuously. This leads to the reducing of the number of antibiotics at the market. 

Microorganisms produce a variety of bioactive compounds that are of scientific and economic interest. For example, they produce a great variety of saturated and unsaturated fatty acids, which can be used as food supplements or in the production of biodiesel. Increasing demand for these products augment the interest for the screening of several microorganisms. Microalgae and cyanobacteria offer numerous advantages for such investigations because of their enormous biodiversity, fast growth rate [19] and occurring in a variety of habitats [20,21,22,23].

The distribution of novel compounds with antibacterial, antiviral, antifungal and antialgal properties was intensively investigated in microalgae and cyanobacteria over the last years [24,25,26,27,28,29]. The production of secondary metabolites with antimicrobial affects was also studied using macroalgae grown under different conditions [30]. Result of these investigations showed that algae are able to produce a large number of compounds with bioactive activities. Dermatological and cosmetic active ingredients with exceptional antibacterial, antifungal, and antiviral properties are extracted from cyanobacterium *Spirulina* and commercially exploited (company Ocean Pharma GmbH from Reinbek, Germany; company of the year 2012 and product of the year 2013 by the journal “Aesthetic Dermatology”). Due to the enormous diversity of microalgae and cyanobacteria, a large number of species has not been investigated yet and our current understanding on the distribution of bioactive compounds is far from being complete.

### 1.3. Aims of the Study

In the present study, 45 cyanobacterial and microalgal strains from five different divisions, eight classes and one unclear taxon were screened for their potential use in the production of biomass and biogas. Furthermore we investigated a total of 97 microalgal as well as cyanobacterial strains from five different divisions, ten classes, one unclear taxon and one unrevised taxon against three bacteria (*Bacillus subtilis* DSM 10*, Escherichia coli* DSM 18039 and *Pseudomonas fluorescens* DSM 50090) and two fungi (*Candida albicans* DSM 1386 and *Saccharomyces cerevisiae* S 150-2b ATCC 96686) for the distribution of antibacterial and antifungal compounds. The aim of these investigations was to find the best microalgae and cyanobacteria suited for biomass and biogas production as well as bioactive compound and to test if the production depends on phylogenetic relationship at division level or between species of the same genus or even among multiple isolates of the same species

## 2. Materials and Methods

### Organisms and Culture Conditions

Most strains investigated in this study were obtained from the culture collection of algae at the Goettingen University (SAG), Germany [31]. One strain was obtained from the culture collection of algal laboratory (CCALA), Czech Republic [32]. *Scenedesmus* sp. Kiel was isolated from an open pond at the University of Kiel in Kiel, Germany and *Klebsormidium* sp. Namibia 5 was isolated on surface of concrete wall in Namibia. The cultivation was carried out in 400 mL culture vessels placed in a modified Kniese apparatus and 10 l glass bottles in liquid culture medium under continuous aeration with filtered air for the investigation of antibacterial and antifungal compounds as well as biogas production, respectively. Liquid media 3NBBM+V (modified Bold’s Basal Medium), ASM 15 and 30 (Artificial Seawater Medium) [33] and Spirulina Medium [34] were used for the cultivation. Microalgal and cyanobacterial cultures were grown on a 14:10 hour light:dark cycle at 135 µmol photons m^−2^ s^−1^ of white fluorescent light at 21 °C or 26 °C, respectively. The harvesting was done by centrifugation at 12,851 g for 10 minutes. For the investigation of biogas production the pellets were stored at −20 °C until further processing. For the investigation of antibacterial and antifungal compounds the obtained pellets were freeze-dried. The supernatant was collected in new tubes. Both the supernatant collected and the freeze-dried pellets were stored at −20 °C until analysis. Microscopy was done to check if the cultures were axenic (Leica DC 300F, Serien No. 14710203, Leica Microsystems AG, Heerbrugg).

## 3. Analytical Methods

### 3.1. Growth Analysis

Dry weight (DW) was analyzed to determine the biomass production. For the determination of dry weight, 5 mL of culture were filtered on a preweighted Whatman filter GF/C (1.2 µm, diameter 47 mm). The filters were subsequently kept in an incubator at 105 °C overnight and then weighed. Optical density (OD) was measured with a UV-2501 PC Photometer (Shimadzu, Kyoto, Japan) at a wavelength of 750 nm to determine the correlation between the culture density and the production of bioactive compounds. A pulse amplitude-modulated chlorophyll fluorometer (Mini PAM, Heinz Walz, Effeltrich, Germany) was used for determination of the effective quantum yield of photosystem II (Fv'/Fm') to control the physiological state of the cultures.

### 3.2. Determination of Dry Mass (DM) and Organic Dry Mass (ODM)

Measurements of dry mass (DM) and organic dry mass (ODM) were conducted according to VDI guideline 4630 [35]. 2g to 3g of biomass were filled in crucibles of known dry weight, weighed to determine the fresh mass content (FM) and treated in a drying oven until constant weight (ca. 24 h). After that and cooling in a desiccator the dry mass was determined by weighing and taking ratio of:

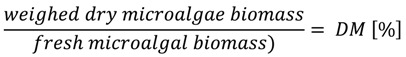
(1)


After the determination of the DM the same crucibles were treated in a furnace at temperatures of more than 600 °C until constant weight (*ca.* 6 h). After that and cooling in a desiccator the dry mass was determined by weighing the ash and taking ratio of:

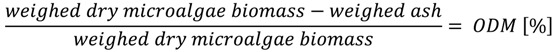
(2)


### 3.3. Measuring Biogas Production

Measurements were conducted according to VDI guideline 4630 [35]. According to VDI4630 sewage sludge was used as inocculum. 300 g of inocculuum was filled in a bottle. Microalgae biomass was added to the inocculum. The mixture concentration was based on the organic dry mass (ODM) content by taking ratio of:

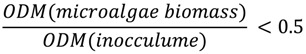
(3)


Inocculum without microalgae biomass was used as a reference. The digester bottle was placed for 30 days in a water bath where the water is maintained at a temperature of 38 °C. The digester bottle was gas-tightly linked with bottle containing 500 mL water. The last was connected with a graduated cylinder. The graduated cylinder was covered with parafilm in order to avoid the evaporation of the collected water. The volume of the biogas produced under anaerobic condition was determined by the measurement of water displaced in the graduated cylinder.

### 3.4. Productivity of Biogas

The biomass dry weight (DW) and biogas yield (BY) were used to calculate productivity (P). They were implemented in the following equation:

*P* = *DW* × *BY* (4)

Where P (ml/l culture volume) is the productivity, DW (g/l DW) is the biomass production, BY (ml/g ODM) is the biogas yield. According to standard measurement procedures, like VDI 4630, the total biogas volumes of all gaseous components except humidity were calculated for normal pressure (p = 101,325 Pa) and normal temperature (273.15 K) conditions [35].

### 3.5. Extraction of Bioactive Compounds

To the freeze-dried biomass (*ca.* 100 mg), 1 mL of 100% methanol was added. The suspension was shaken for 15 minutes (Multi-Tube Vortex Mixer, Bender und Hobein AG, Serien No. 5693, Zürich) and centrifuged at 12,851 g for 10 minutes at 4 °C. The methanol extracts were dried under a gentle stream of nitrogen. The dried extracts were dissolved in 400 µL of 50% of methanol.

### 3.6. Bacterial and Fungal Organisms Used and Culture Conditions

Microorganisms investigated in this study were obtained from the German Collection of Microorganisms and Cell Cultures (DMSZ), Germany [36]. *Saccharomyces cerevisiae* S 150-2b was obtained from the American Type Culture Collection (ATCC) [37]. Two gram-negative bacteria (*Escherichia coli* DSM 18039 and *Pseudomonas fluorescens* DSM 50090), one gram-positive bacterium (*Bacillus subtilis* DSM 10), and two yeast bacteria (*Candida albicans* DSM 1386 and *Saccharomyces cerevisiae* S 150-2b ATCC 96686) were cultivated for agar diffusion tests. Lysogeny-broth-Medium, YPD medium and the universal yeast medium M 186 were used for the cultivation of bacteria, *Saccharomyces cerevisiae* and *Candida albicans*, respectively. An amount of 200 µL of bacterial or fungal suspension was applied onto the surface of the plate with agar medium. According to the method of Cannell *et al.* [38], wells of 5 mm diameter were bored in the plates and filled with either methanol extracts, culture supernatant, pellet, 50% methanol (as negative control) or antibiotics (as positive control). The agar plates with bacteria or fungi were incubated overnight at 37 °C and 28 °C, respectively, after the plates were preincubated at 4 °C for 2 h. The latter incubation was done to prevent evaporation of the methanol and to allow diffusion of the extracts into the agar.

### 3.7. Determination of Optimal Antibiotic Concentration

For the agar-well diffusion test, two antibiotics Chloramphenicol and Erythromycin were used as positive controls to inhibit the growing of *P. fluorescens* as well as *E. coli* and *B. subtilis* respectively. Different antibiotic concentrations (0.06, 0.12, 1.5, 4, 6, 12 mg/L) were tested to determinate the optimal effect. If the antibiotic was effective against bacteria at a certain concentration and had a greater zone of inhibition, this zone of inhibition was measured and considered as 100% inhibition. Inhibition produced by methanol extracts, culture supernatant, pellet, 50% methanol was compared with that produced by effective concentration of antibiotics.

## 4. Results and Discussion

### 4.1. Biomass Production of Microalgal and Cyanobacterial Strains

Results of the biomass production of microalgal and cyanobacterial cultures are shown in Table 1. The lowest biomass production was observed by *Sphaerosorus composita* SAG 53.91, *Trebouxia showmanii* SAG 2009 and *Scenedesmus obliquus* SAG 276-1 with 1.00 ± 0.04 g/L DW, 1.18 ± 0.18 g/L DW and 1.27 ± 0.22 g/L DW, respectively. *Arthrospira maxima* SAG 84.79, *Chlorella vulgaris* SAG 211-11b, *Chlorella stigmatophora* SAG 9.86 und *Porphyridium sordidum* SAG 44.94 showed the highest biomass production of 5.72 ± 0.18 g/L DW, 4.85 ± 0.15 g/L DW, 4.63 ± 0.42 g/L DW and 4.12 ± 0.03 g/L DW, respectively. The ability to produce the biomass was different by the strains because they are from various ecological niches. Under environmental conditions algae are adapted to different light conditions and growth temperatures as well as they may additional utilize during their growth different organic substances. Standardized culture conditions could be close to the optimum for some algal strains but at the same time for others they are close to their survival borders. For example, *Sphaerosorus composita* SAG 53.91 was isolated from shell found in Kieler Förde in 12 m water depth so one may expect that standard light in our experiment was too bright as well as growth temperature was too high (as probably also for other Xanthophyceae algae which are known often to be cold tolerant); other badly growing strain *Trebouxia showmanii* SAG 2009 under environmental conditions was growing as phycobiont of lichen and may need some vitamins or other organic substances to increase growth rate. Thus for mass production culture conditions need to be adjusted for each strain to get the highest biomass production. Our results are similar to the biomass production reported for *Chlorella vulgaris* by Degen *et al.* [39], *i.e.*, with a value of about 4.8 g/L DW. The highest biomass production obtained in this study is lower than the biomass production reported for *Chlorella zofingiensis* CCAP 211/14, *Muriellopsis aurantiaca* SAG 249-1, *Muriellopsis decolor* SAG 249-2 and *Neospongiococcum gelatinosum* SAG B 64.80 by Del Campo *et al.* [40], *i.e.*, with values of 6.71 ± 0.5 g/L DW, 6.73 ± 0.6 g/L DW, 9.60 ± 1.1 DW and 6.78 ± 0.8 g/L DW, respectively. However, these values have been obtained for cultures aerated with 1% CO_2_. In our previous study, 5% (v/v) CO_2_ has been shown to result in significantly increased algal biomass production compared to gassing with air [9]. These authors showed that the biomass production increased from 1.31 ± 0.11 g/L DW to 7.14 ± 0.16 g/L DW for *Coccomyxa* sp. SAG 2391, from 3.15 ± 0.21 g/L DW to 7.34 ± 0.09 g/L DW for *Desmodesmus* sp. SAG 2389 and from 2.57 ± 0.19 g/L DW to 7.23 ± 0.06 g/L DW for *Muriella terrestris* SAG 2390. Thus the already high production of biomass obtained in this study might even be increased if CO_2_ gassing would be applied.

### 4.2. Screening for Microalgal and Cyanobacterial Biogas Production and Productivity

Biogas yields of different microalgal and cyanobacterial species are reported in Table 1. The biogas yields varied from 202 to 576 mL/g ODM and are in the range of previously published data [14,16,41,42]. 

**Table 1:** Biogas yield (BY) (mL/g ODM), biomass production (DW) (g/l DW) and biogas productivity (P) (mL/l culture volume) of microalgae and cyanobacteria strains. Maize starch was used as a positive control. Sewage sludge without substrate addition was used as a reference and was subtracted.

Division/Class	Genus/Species	SAG Strain No	Culture Medium	Biogas yield (BY)	Biomass dry mass (DW)	Biogas productivity (P)
Chlorophyta						
Chlorophyceae	*Desmodesmus.* sp.	2389	3NBBM	456	3.15±0.21	1436.40
	*D. armatus*	276–4e	3NBBM	440	3.00±0.43	1318.53
	*D. armatus*	276–4d	3NBBM	518	2.55±0.75	1319.17
	*Haematococcus. pluvialis*	44.96	3NBBM	300	2.03±0.27	610.00
	*H. pluvialis*	192.80	3NBBM	413	3.05±0.77	1261.03
	*Scenedesmus.* sp.	Kiel*	3NBBM	373	2.00±0.07	746.00
	*S. obliquus*	276–1	3NBBM	433	1.27±0.22	548.47
Chlorodendrophyceae	*Tetraselmis striata*	41.85	3NBBM	385	2.91±0.16	1121.63
Trebouxiophyceae	*Chlorella sorokiniana*	211–8k	3NBBM	320	1.43±0.11	456.53
	*C. vulgaris*	211–11b	3NBBM	410	4.85±0.15	1987.13
	*C. vulgaris*	211–1e	3NBBM	510	3.81±0.07	1944.80
	*C. vulgaris*	211–8l	3NBBM	436	3.00±0.05	1306.55
	*C. vulgaris*	211–11f	3NBBM	428	3.88±0.13	1660.64
	*C. vulgaris*	211–8m	3NBBM	463	3.64±0.06	1683.78
	*C. vulgaris*	211–11s	3NBBM	364	3.86±0.07	1406.25
	*C. vulgaris*	2.80	3NBBM	397	3.66±0.10	1454.34
	*C. vulgaris*	9.88	3NBBM	431	3.70±0.23	1594.70
	*Chloroidium. saccharophilum*	2149	3NBBM	304	1.63±0.08	495.52
	*C. saccharophilum*	56.87	3NBBM	303	2.93±0.11	887.79
	*Geminella.* sp.	57.90	3NBBM	448	2.19±0.39	981.12
	*Geminella* sp.	49.80	3NBBM	440	1.92±0.15	843.33
	*G. minor*	22.88	3NBBM	376	2.75±0.14	1032.74
	*G. terricola*	20.91	3NBBM	316	1.42±0.35	449.77
	*Nannochloris.* sp.	251–2	3NBBM	494	2.41±0.18	1192.19
	*Stichococcus.* sp.	2118	3NBBM	576	2.40±0.06	1384.32
	*Trebouxia. showmanii*	2009	3NBBM	461	1.18±0.18	542.44
unclear taxonomy	*Chlorella stigmatophora*	9.86	3NBBM	380	4.63±0.42	1761.93
Heterokontophyta						
Eustigmatophyceae	*Chloridella. neglecta*	48.84	3NBBM	380	2.82±0.18	1071.60
	*Eustigmatos. magnus*	36.89	3NBBM	405	2.66±0.13	1077.30
	*Monodus. unipapilla*	8.83	3NBBM	217	1.75±0.07	379.03
	*Nannochloropsis. salina*	40.85	ASM30	202	3.35±0.05	677.37
	*Vischeria. punctata*	887–1	3NBBM	464	2.26±0.06	1047.09
Xanthophyceae	*Botrydiopsis intercedens*	806–3	3NBBM	235	2.18±0.05	512.30
	*Bumilleriopsis. filiformis*	809–2	3NBBM	353	2.24±0.04	789.54
	*Heterococcus. viridis*	835–7	3NBBM	411	1.72±0.08	706.92
	*Sphaerosorus. composita*	53.91	3NBBM	399	1.00±0.04	399.00
	*Xanthonema. debile*	2289	3NBBM	421	1.73±0.14	726.93
	*X. sessile*	2193	3NBBM	238	2.28±0.05	541.85
Streptophyta						
Klebsormidiophyceae	*Klebsormidium.* sp.	Namibia 5*	3NBBM	308	2.11±0.03	649.88
Rhodophyta						
Porphyridiophyceae	*Porphyridium. purpureum*	1380–1d	ASM15	264	3.98±0.03	1050.72
	*P. sordidum*	44.94	ASM15	236	4.12±0.03	973.11
Cyanobacteria						
Cyanophyceae	*Arthrospira. platensis*	86.79	Spirul	376	2.93±0.09	1102.93
	*A. platensis*	21.99	Spirul	395	3.95±0.06	1560.25
	*A. maxima*	84.79	Spirul	260	5.72±0.18	1488.07
	*Oscillatoria* sp.	76.79	Spirul	356	3.31±0.17	1178.36

The biogas yields varied within the range of 300 and 518 mL/g ODM in the green algal class Chlorophyceae and were of similar magnitude (e.g., 303 and 576 mL/g ODM) in the green algal class Trebouxiophyceae. The biogas production is low within the red algal class Porphyridiophyceae with yields of 236 and 264 mL/g ODM, respectively. Based on our results, the green algal classes Chlorodendrophyceae (385 mL/g ODM), Klebsomidiophyceae (308 mL/g ODM), the brown algal classes Eustigmatophyceae (from 202 to 464 mL/g ODM), Xanthophyceae (from 235 to 421 mL/g ODM) and the cyanobacterial class Cyanophyceae (from 260 to 395 mL/g ODM) can be considered as intermediate classes for the production of biogas. *Desmodemus armatus* SAG 276-4d (518 mL/g ODM), *Chlorella vulgaris* (510 mL/g ODM) and *Stichococcus* sp. SAG 2118 (576 mL/g ODM) belonging to the division Chlorophyta expressed the highest yields of biogas production.

Multiple strains of *Chlorella vulgaris* Beijerinck were investigated in order to compare the different ability of biogas production among them. The biogas production of eight strains of *Chlorella vulgaris* was highly variable and ranged from 364 to 510 mL/g ODM. Literature data, where *Chlorella vulgaris* was anaerobically digested, were also variable. This concurs with findings by Müller *et al.* [43], who found considerable genomic variation among the multiple strains of Chlorella vulgaris available from public culture collections. When compared to the existing literature data, our results on biogas production by microalgae are higher those obtained by Ras *et al.* [15], and lower than the theoretical methane potential obtained by Sialve *et al.* [17]. The biogas yields obtained in this study were compared to the methane yields obtained in other studies because we know that the proportion of methane in the biogas produced vary from 69 to 75% for the majority of the studies, regardless of species and operating conditions [17].

Very low yields of 202 mL/g ODM was found for *Nannochloropsis salina* SAG 40.85. This result is similar to the previous study, in which *Nannochloropsis salina* produced low biogas (about 220 mL/g ODM) [44]. Schwede *et al.* [44], observed that the biogas yields of *Nannochloropsis salina* increased about three times after thermal pretreatment compared to the untreated sample. Thus the low biogas yield of our studied strain may have even increased when thermal pretreatment was applied. 

*A. platensis* SAG 86.79 (376 mL/g ODM), *A. platensis* SAG 21.99 (395 mL/g ODM) and *Oscillatoria* sp (356 mL/g ODM) expressed a significantly high biogas productivity compared to *A. maxima* SAG 84.79 (260 mL/g ODM). The biogas production of cyanobacteria is thus similar to those of Heterokontophyta. The biogas yields of our cyanobaterial strains were lower than the theoretical methane yields (470–740 mLCH_4_ /g VS) obtained by Sialve *et al.* [17]. 

The potential of substrates to produce biogas depends on the biodegradability of the synthesized organic compounds. For microalgae and cyanobacteria, the cell membrane plays an important role in the protection of the cell interior against the attack of anaerobic bacteria. Algae (especially from different classes) vary in their cell wall structure and its chemical composition. They may contain cellulose, pectin, and other compounds differently arranged and in different proportions [45]. 

Other important difference is that algae from different algal classes have different storage products. Similar to higher plants, green algae accumulate starch in their plastids. Red algae accumulate starch granules outside of their plastids. The starch granules from red algae (also known as floridean starch) show structural similarities with higher plant starch granules but lack amylose [46]. Heterokont algae do not produce starch at all, but accumulate oils.

The most common storage products of cyanobacteria are polyphosphate as a phosphorus storage compound, cyanophycin or phycobilin protein pigment as nitrogen storage products, and glycogen as a storage product of both carbon and energy [47].

Cultures growing under optimal conditions could accumulate less reserve substances compared to cultures, which grow under unfavorable conditions. Different pretreatments applied on a small number of mono-, as well as multispecific, microalgal and cyanobacterial biomass are known to enhance biogas production. The influence of thermochemical pretreatment was used on multispecific biomass collected from the effluent of high-rate sewage stabilization ponds [48]. This study has shown that temperature is the most important parameter; duration is the second most important parameter; and concentration is the least important parameter in the pretreatment process of algal fermentation. Likewise, Schwede *et al.* [44], studied the effects of thermal pretreatment on anaerobic digestion of *Nannochloropsis salina* biomass and found that the pretreatments at 100 and 120 °C with 2 or 8 h pretreatment time or pressure compensation after the heating period significantly increased the biogas and methane yields. Mussgnug *et al.* [14] demonstrated that drying of algal biomass at 105 °C for 24 h as a pretreatment decreased the amount of biogas production to ca. 80% and hydrogen production in *Chlamydomonas reinhardtii* prior to anaerobic fermentation of the algae biomass increased the biogas production to 123%. Due to the enormous biodiversity of microalgae and cyanobacteria and the variety of their cell membrane, additional studies of the effect of pretreatments of microalgae and cyanobacteria biomass are needed to optimize the anaerobic cell degradability, and, therefore, to augment the biogas production.

The biogas yield and biomass production were taken into account to calculate biogas productivity. The highest productivities were obtained in *Chlorella vulgaris* strains SAG 211-11b and SAG 211-11e with 1987.13 mL/L culture volume and 1944.80 mL/L culture volume, respectively. Both *Chlorella vulgaris* strains had high biomass production (4.85 ± 0.15 g/l DW and 3.81 ± 0.07 g/L DW) and biogas yields (410 mL/g ODM and 510 mL/g ODM). The productivity of both *Chlorella vulgaris* strains were thus considerably higher than the one of *Stichococcus* sp. SAG 2118 (1384.32 mL/L culture volume), which had the highest biogas yield (576 mL/g ODM) obtained in this study but a lower biomass production (2.40 ± 0.06 g/L DW). In contrast, *Chlorella stigmatophora* SAG 9.86 had a low biogas yield (380 mL/g ODM) but a high biomass production (4.63 ± 0.42 g/L DW) and showed the third highest productivity (1761.93 mL/L culture volume) obtained in this study (Table 1).

### 4.3. Screening for Microalgal and Cyanobacterial Bioactivities Against Bacteria and Fungi

The antibacterial and antifungal effects of microalgae and cyanobacteria are shown in Table 2. The 97 strains analyzed in this study belonged to four major divisions of microalgae and one cyanobacterial division. The microalgae division Chlorophyta had a high number of species that belonged to the classes Trebouxiophyceae, Chlorophyceae, Chlorodendrophyceae and unclear taxon with 33, 24, 3, and 2 strains, respectively. The division Heterokontophyta contained two classes, namely *Eustigmatophyceae* and *Xanthophyceae* with 9 and 6 strains, respectively. One strain of the class Klebsormidiophyceae and one strain of the class Zygnematophyceae represented by the division Streptophyta were analyzed. The divisions Rhodophyta was represented by 7 and 2 strains belonging to the classes Porphyridiophyceae and Rhodophyceae, respectively. The division Cyanobacteria contained one class, namely Cyanophyceae, and unrevised taxon with 8 and 1 strain, respectively.

All cultures were first investigated by light microscopy to ensure that they were axenic. Results of this screening showed that 82 of the 97 analyzed cultures were without contamination. The remaining 15 cultures were not axenic and contained either bacterial or some other type of contamination (Table 2).

**Table 2:** Antibacterial and antifungal effects from microalgae and cyanobacteria against *Bacillus subtilis* DSM 10 (B), *Escherichia coli* DSM 18039 (E), *Pseudomonas fluorescens* DSM 50090 (P), *Candida albicans* DSM 1386 (C) and *Saccharomyces. cerevisiae* S 150–2b ATCC 96686 (S). Activities are given in percent (%) for bacteria as well as + (< 10 mm diameter) and ++ (≥ 10 mm diameter) for fungi and—no inhibition.

Division/Class	Genus/Species	SAG Strain No	Culture Medium	Axenic culture	Methanol extract	Pellet	supernatant	Degree of inhibition in %
Chlorophyta								
Chlorophyceae	*Bracteacoccus sp.*	2137	3NBBM	yes	P	-	-	40
	*B. bullatus*	2032	3NBBM	yes	-	-	-	
	*Chromochloris cinnabarinus*	221–2	3NBBM	yes	-	-	-	
	*Coleochlamys oleifera*	6.90	3NBBM	yes	-	-	B	5
	*Desmodesmus. *sp.^b^	2389	3NBBM	yes	-	-	-	
	*D. armatus*^b^	276–4e	3NBBM	yes	E	-	-	10
	*Haematococcus pluvialis*^b^	44.96	3NBBM	yes	-	B	-	20
	*H. pluvialis*	34–1a	3NBBM	yes	-	B	-	20
	*H. pluvialis*	34–1b	3NBBM	yes	-	-	-	
	*Neospongiococcum gelatinosum*	64.80	3NBBM	yes	B	-	-	40
	*Pectinodesmus. pectinatus*	2003	3NBBM	yes	-	-	-	
	*Scenedesmus* sp.	2125	3NBBM	no	-	-	-	
	*Scenedesmus* sp.^b^	Kiel*	3NBBM	yes	-	-	-	
	*S. acuminatus*	38.81	3NBBM	yes	B	B	B	40/20/20
	*S. costatus*	46.88	3NBBM	yes	B	B	B	20/20/20
	*S. falcatus*	2.81	3NBBM	yes	B	B	B	40/20/20
	*S. ovalternus*	52.80	3NBBM	yes	B	B	B	20/20/20
	*S. obliquus*^b^	276–1	3NBBM	yes	-	-	-	
	*S. pectinatus*	2003	3NBBM	yes	B	-	B	40/20
	*S. rubescens*	5.95	3NBBM	yes	-	-	-	
	*S. wisconsinensis*	22.81	3NBBM	yes	B	B	B	40/40/40
	*Scotiellopsis. oocystiformis*	277–1	3NBBM	yes	-	-	-	
	*Tetraedron caudatum*	2092	3NBBM	yes	B	-	-	20
	*T. minimum*	44.81	3NBBM	yes	B	-	-	20
Chlorodendrophyceae	*Tetraselmis* sp.	3.98	3NBBM	yes	B	B	B	40/40/20
	*T. striata*^b^	41.85	3NBBM	yes	B	B	B	20/20/20
	*T. tetrathele*	161–2b	3NBBM	yes	-	-	-	
Trebouxiophyceae	*Chlorella mirabilis*	38.88	3NBBM	yes	-	-	-	
	*C. sorokiniana*	211–31	3NBBM	yes	-	-	-	
	*C. sorokiniana*	211–32	3NBBM	yes	-	-	E	40
	*C. sorokiniana*^b^	211–8k	3NBBM	yes	-	-	-	
	*C. vulgaris*^b^	211–11b	3NBBM	yes	-	-	-	
	*Chloroidium. ellipsoideum*	2140	3NBBM	yes	-	-	-	
	*C. angustoellipsoideum*	2041	3NBBM	yes	-	-	-	
	*C. saccharophilum*^b^	2149	3NBBM	yes	-	-	B	20
	*C. saccharophilum*^b^	56.87	3NBBM	yes	-	-	-	
	*Coccomyxa.* sp.	2391	3NBBM	yes	-	-	-	
	*C. elongata*	216–3a	3NBBM	yes	P	-	-	5
	*Coenocystis.* sp.	LS5-R4*	3NBBM	yes	-	-	-	
	*Diplosphaera. mucosa*	48.86	3NBBM	yes	-	-	-	
	*Geminella* sp.^b^	57.90	3NBBM	yes	E/B	-	-	5/10
	*G. minor*	10.91	3NBBM	yes	P	-	-	20
	*G. terricola*^b^	20.91	3NBBM	yes	P	-	-	10
	*Heterochlorella luteoviridis*	211–2a	3NBBM	yes	-	-	-	
	*H. luteoviridis*	211–3	3NBBM	yes	-	-	C	+
	*Muriella. terrestris*	2390	3NBBM	yes	-	-	-	
	*Nannochloris eucaryotum*	55.87	3NBBM	no	-	-	-	
	*N. normandinae*	9.82	3NBBM	no	-	-	-	
	*Neocystis. mucosa*	40.88	3NBBM	yes	-	-	-	
	*Pabia signiensis*	7.90	3NBBM	yes	-	-	B	5
	*Pseudochlorella. pringsheimii*	211–1a	3NBBM	yes	-	-	-	
	*Raphidonema* sp.	LS11-R7A*	3NBBM	yes	P	-	-	5
	*Stichococcus* sp.	249.80	3NBBM	yes	-	-	-	
	*Stichococcus* sp.	2059	3NBBM	yes	-	-	-	
	*S. bacillaris*	379–1b	3NBBM	yes	-	B	-	40
	*S. bacillaris*	379–2	3NBBM	yes	-	-	-	
	*S. deasonii*	2139	3NBBM	yes	-	-		
Chlorophyta								
Trebouxiophyceae	*Trebouxia. aggregata*	219–1d	3NBBM	yes	-	-	-	
	*T. asymmetrica*	48.88	3NBBM	yes	-	-	-	
	*T. showmanii*^b^	2009	3NBBM	yes	-	-	-	
unclear taxonomy	*Chlorella stigmatophora*^b^	9.86	3NBBM	no	-	-	-	
	*Chlorella* sp.	15.93	3NBBM	yes	-	-	-	
Heterokontophyta								
Eustigmatophyceae	*Chloridella. neglecta*	7.88	3NBBM	yes	P	-	-	25
	*C. simplex*	51.91	3NBBM	yes	-	-	-	
	*Eustigmatos magnus*^b^	36.89	3NBBM	yes	-	-	-	
	*Monodus. unipapilla*^b^	8.83	3NBBM	yes	-	-	-	
	*Nannochloropsis. gaditana*	2.99	ASM15	no	-	-	-	
	*N. salina*^b^	40.85	ASM30	yes	-	-	-	
	*Vischeria. helvetica*	876–1	3NBBM	yes	-	B	-	40
	*V. stellata*	33.83	3NBBM	yes	-	-	-	
	*V. stellata*	887–2	3NBBM	yes	-	B	-	20
Xanthophyceae	*Botrydiopsis alpina*	CCALA 217*	3NBBM	yes	-	-	-	
	*B. intercedens*^b^	806–3	3NBBM	yes	-	-	-	
	*Bumilleriopsis. filiformis* ^b^	809–2	3NBBM	yes	P	-	-	20
	*Sphaerosorus composita* ^b^	53.91	3NBBM	yes	-	-	-	
	*Xanthonema. exile*	2286	3NBBM	no	P	-		5
	*X. sessile* ^b^	2193	3NBBM	no	-	-	-	
Streptophyta					-	-	-	
Klebsormidiophyceae	*Klebsormidium* sp.^b^	Namibia 5*	3NBBM	yes	B	B		40/70
Zygnematophyceae	*Fottea pyrenoidosa*	1.88	3NBBM	yes	-	-	-	
Rhodophyta					-	-	-	
Porphyridiophyceae	*Porphyridium. purpureum*	113.79	ASM15	no	E	-	-	40
	*P. purpureum*	1380–1a	ASM15	yes	-	-	-	
	*P. purpureum*	1380–1b	ASM15	yes	-	-	-	
	*P. purpureum*	1380–1c	ASM15	yes	E/B	-	-	60/30
	*P. purpureum*^b^	1380–1d	ASM15	yes	-	-	C	+
	*P. purpureum*	1380–1f	ASM15	yes	E/B	-	-	40/10
	*P. sordidum*^b^	44.94	ASM15	no	-	E/B/P	-	40/20/40
Rhodellophyceae	*Rhodella. maculata*	45.85	ASM15	no	B	B	B	20/40/40
	*R. violaceae*	115.79	ASM15	yes	B	B	-	40/40
Cyanobacteria					-	-	-	
Cyanophyceae	*Arthrospira platensis*	85.79	Spirul	yes	S	-	-	++
	*A. platensis*^b^	86.79	Spirul	no	-	-	-	
	*A. platensis*	257.80	Spirul	yes	E/B	-	-	40/5
	*A. platensis*^b^	21.99	Spirul	yes	-	-	-	
	*A. maxima*^b^	84.79	Spirul	no	S	-	-	+
	*A. maxima*	49.88	Spirul	no	-	-	-	
	*Oscillatoria* sp.	50.96	ASM30	yes	-	-	-	
	*Oscillatoria.* sp.^b^	76.79	Spirul	no	B	B	B	30/50/40
unrevised taxon	„Spirulina“ laxissima	256.80	Spirul	no	E	-	-	40

^b^ Strains for biogas analysis were marked with.

The effective quantum yield of photosystem II (Fv'/Fm') was determined in all cultures before harvesting. Results of these measurements showed a good physiological status (photosynthetic performance) of the investigated microalgae and cyanobacteria. OD at a wavelength of 750 nm was measured to control the growth of cultures. The harvesting was done during the fourth week of growth representing the stationary phase.

Of the analyzed 97 strains, a total of 44 strains showed inhibition against bacteria or fungi. That means that 45% of all strains investigated in this study synthesizing bioactive compounds. Antibacterial effects were detected in 40 strains, whereas 23 strains were only active against *B. subtilis,* 8 strains against *P. fluorescens* and 4 strains against *E. coli*. Four strains were active against both *B. subtilis* and *E. coli.* Only one strain, *Porphyridium sordidum* SAG 44.94, showed inhibitions against all three bacteria analyzed. Antifungal effects were detected in only four strains, *Heterochlorella luteoviridis* SAG 211-3, *Porphyridium purpureum* SAG 1380-1d, *Arthrospira platensis* SAG 85.79 and *A. maxima* SAG 84.79. *H. luteoviridis* SAG 211-3 and *P. purpureum* SAG 1380-1d were active against *C. albicans*. *A. platensis* SAG 85.79 and *A. maxima* SAG 84.79 showed inhibition against *S. cerevisiae.*

The methanol extracts of 32 strains showed antibacterial and antifungal effects. The methanol extracts of 15 strains were active against *B. subtilis*, 8 against *P. fluorescens*, 3 against *E. coli,* 4 against both *B. subtilis*, as well as *E. coli* and 2 against *S. cerevisiae.* No activity was found against *C. albicans.* Antibacterial activities were detected in the pellets of 17 strains with pellets of 16 strains being active against *B. subtilis* and the pellet of *P. sordidum* SAG 44.94 being active against *B. subtilis,* *E. coli* and *P. fluorescens.* No antifungal activities were observed in any of the pellets. Antibacterial and antifungal activities were also found in the aqueous extracellular supernatants. Antibacterial effects were found in the supernatants of 14 strains against *B. subtilis* and *E. coli*, respectively. Antifungal activity against *C. albicans* was detected in the supernatant of *Heterochlorella luteoviridis* SAG 211-3 and *Porphyridium purpureum* SAG 1380-1d. 

The antibacterial activity against *B. subtilis* varied from 5% to 70%, whereas 5% inhibition was observed in 3 tests, 10% inhibition in 2 tests, 20% inhibition in 23 tests, 30% inhibition in 1 test, 40% inhibition in 17 tests, 50% inhibition in 1 test and 70% inhibition in 1 test compared with the zone of inhibition of Erythromycin 6 mg/mL (30 mm of diameter) that was considered as 100%. The antibacterial activity against *E. coli* varied from 5% to 60%, whereas 5% inhibition was detected in 1 test, 10% inhibition in 1 test, 40% inhibition in 6 tests and 60% inhibition in 1 test compared with the zone of inhibition of Erythromycin 6 mg/mL (25 mm of diameter) that was considered as 100%. The antibacterial activity against *P. fluorescens* varied from 5% to 40%, whereas 5% inhibition was detected in 3 tests, 10% inhibition in 1 test, 20% inhibition in 2 tests, 25% inhibition in 1 test and 40% inhibition in 2 tests compared with Chloramphenicol 4 mg/mL (25 mm of diameter) that was considered as 100%. The antifungal activity was estimated with + + for a zone of inhibition greater than 10 mm of diameter or + for a zone of inhibition of equal or smaller than 10 mm of diameter due to the fact that no antibiotic was used as reference. The antifungal effect against *C. albicans* was evaluated with + in two tests and against *S. cerevisiae* with + and ++ in two tests.

The ability of microalgae and cyanobacteria to produce bioactive compounds was already reported in numerous other studies [24,25,38,49]. However, due to the enormous biodiversity of microalgae and cyanobacteria, further investigations are needed. With 45% of all strains investigated in this study synthesizing bioactive compounds, the results of our screening is significantly higher than those observed in previous studies such as those by Flores and Wolk [49], Cannell *et al.*, [38] and Patterson *et al.* [24] with 11%, 10%, and 7%, respectively. 

Our study shows that the capability to produce antibacterial and antifungal compounds has evolved independent of phylogenetic relationship in microalgae and cyanobacteria. It is possible that these compounds are present in some of the investigated microalgae and cyanobacteria but in such small quantities that they could not be detected. However, our results demonstrate that a greater number of microalgae and cyanobacteria contain bioactive compounds. Indeed, we observed antibacterial effects against *B. subtilis* by a great majority of microalgae analyzed in this study. In contrast, inhibition against the fungis *C. albicans* and *S. cerevisiae* was rarely detected. These results are in agreement with those of other studies, in which microalgae and cyanobacteria were screened to detect antibacterial effect as well as macroalgae for antimicrobial activities [30,38].

The highest bioactive effect obtained was inhibition of 70 % against the gram-positive bacteria *B. subtilis* in *Klebsormidium* sp. The second highest bioactive effect detected was inhibition of 60 % against the gram-negative bacterium *E. coli* in *Porphyridium purpureum* SAG 1380-1C. These high bioactive effects have been obtained by compounds extracted with methanol. Also, the highest antibacterial activity was generally observed in methanol extracts. This result agrees with a previous study, in which different organic solvents were used to extract antibacterial compounds [38]. Debro and Ward [50] used ethanol as extraction solvent and reported results similar to this study. These results reveal that a better part of the bioactive compounds in microalgae and cyanobacteria is hydrophobic and can better be extracted with organic solvents. Our result revealed that methanol extracts exhibited antibacterial activities on both gram-positive and gram-negative bacteria. In contrast, Katircioglu *et al.* [51] found that methanol extracts of various microalgae inhibited the activity of only gram-positive bacteria.

Microalgal pellets investigated in this study expressed only antibacterial activities. Antifungal and antibacterial effects, however, were observed from supernatants of cultures and methanol extracts. Microalgae and cyanobacteria are able to produce substances with bioactive effects and to excrete them in extracellular culture medium. Han *et al.* [52] and Park *et al.* [53] demonstrate the presence of chitinolytic enzymes in culture supernatants of *Chlorella vulgaris* and *Chlamydomonas reinhardtii* that were cultivated at defined conditions. 

Multiple research projects have been performed to investigate the influence of cultivation conditions on the production of bioactive compounds in microalgae and cyanobacteria. Seaweed-type bioreactors were chosen and optimal physical conditions were determined in order to increase the production of antibiotics by the cyanobacterium *Scytonema* sp. TISTR 8208 [54]. Likewise, Bloor and England [55] studied the effect of nutrient factors and found that 26.4 mM nitrate and 6 µM iron were the optimal concentrations for maximizing antibiotic production by the cyanobacterium *Nostoc muscorum.* The effects of temperature and pH on both growth and antimicrobial activities were investigated in the cyanobacterium *Synechococcus leopoliensis.* A temperature of 35 °C and a pH of 8 were the best factors influencing the growth of this cyanobacterium and the production of antimicrobial agents [56]. Cytotoxic, antibacterial and antifungal activities of the cyanobacterium *Gloeocapsa* sp. strain Gacheva 2007/R-06/1 were stimulated at suboptimal temperatures (15–26 °C), low light and prolonged cultivation [57]. Thus, the already high percentage of positive results of our studied strains may even be enhanced when the culture conditions were optimized for the production of antimicrobial agents.

## Conclusions

In this work, 45 cyanobacterial and microalgal strains from five different divisions, eight classes and one unclear taxon were also investigated for their potential use in biomass and biogas production. All strains were grown under the same culture conditions to allow an interspecies comparison. Our results show that the production of biogas by the investigated microalgal and cyanobacterial strains is highly dependent on both taxonomic division and species. The division Chlorophyta contained species that had the highest yield of biogas production. Different pretreatments of biomass are required to increase the biogas production. Furthermore we investigated 97 microalgae and cyanobacteria for antibacterial and antifungal effects. The majority of strains are considered as good candidates for further biochemical and biotechnological analyses in order to characterize and purify novel antimicrobial compounds. 

## Outlook

Some of the strains investigated in this study, such as *Desmodesmus armatus* SAG 276-4e, *Geminella* sp. SAG 57.90, *Tetraselmis striata* SAG41.85, *Bumilleriopsis filiformis* SAG 809-2, *Klebsormidium* sp. Namibia 5, *Porphyridium sordidum* SAG 44.94, *Arthrospira maxima* SAG 84.79 and *Oscillatoria* sp. SAG 76.79 may represent a potential source of substances that are of interest in bioenergy production and have a pharmacological perspective. These strains will be selected for further study focusing on optimizing the processes involved in microalgae and cyanobacteria biorefinery, in which the extraction of bioactive compounds prior to anaerobic fermentation can be considered as pretreatment of microalgal and cyanobacterial biomass in order to increase the anaerobic biodegradability. 

## Author Conrtibutions

Nataliya Rybalka selected and cultivated the strains investigated. Thomas Friedl supervised the selection of the strains and provided strain information. Opayi Mudimu performed the research and analysis. Jens Born supervised and performed the biogas analysis. Rüdiger Schulz provided project ideas and discussion of results as well supervised the research. Opayi Mudimu and Thorsten Bauersachs prepared the manuscript. Opayi Mudimu wrote the article. All authors read and approved the final manuscript.
